# Different decision deficits impair response inhibition in progressive supranuclear palsy and Parkinson’s disease

**DOI:** 10.1093/brain/awv331

**Published:** 2015-11-18

**Authors:** Jiaxiang Zhang, Timothy Rittman, Cristina Nombela, Alessandro Fois, Ian Coyle-Gilchrist, Roger A. Barker, Laura E. Hughes, James B. Rowe

**Affiliations:** ^1^ 1 School of Psychology, Cardiff University, Cardiff CF10 3AT, UK; ^2^ 2 Cognition and Brain Sciences Unit, Medical Research Council, Cambridge CB2 7EF, UK; ^3^ 3 Department of Clinical Neurosciences, University of Cambridge, Cambridge CB2 2QQ, UK; ^4^ 4 Behavioural and Clinical Neuroscience Institute, Cambridge, CB2 3EB, UK

**Keywords:** progressive supranuclear palsy, Parkinson’s disease, saccadic inhibition, drift-diffusion model, Bayesian hierarchical model

## Abstract

Progressive supranuclear palsy and Parkinson’s disease have distinct underlying neuropathology, but both diseases affect cognitive function in addition to causing a movement disorder. They impair response inhibition and may lead to impulsivity, which can occur even in the presence of profound akinesia and rigidity. The current study examined the mechanisms of cognitive impairments underlying disinhibition, using horizontal saccadic latencies that obviate the impact of limb slowness on executing response decisions. Nineteen patients with clinically diagnosed progressive supranuclear palsy (Richardson’s syndrome), 24 patients with clinically diagnosed Parkinson’s disease and 26 healthy control subjects completed a saccadic Go/No-Go task with a head-mounted infrared saccadometer. Participants were cued on each trial to make a pro-saccade to a horizontal target or withhold their responses. Both patient groups had impaired behavioural performance, with more commission errors than controls. Mean saccadic latencies were similar between all three groups. We analysed behavioural responses as a binary decision between Go and No-Go choices. By using Bayesian parameter estimation, we fitted a hierarchical drift–diffusion model to individual participants’ single trial data. The model decomposes saccadic latencies into parameters for the decision process: decision boundary, drift rate of accumulation, decision bias, and non-decision time. In a leave-one-out three-way classification analysis, the model parameters provided better discrimination between patients and controls than raw behavioural measures. Furthermore, the model revealed disease-specific deficits in the Go/No-Go decision process. Both patient groups had slower drift rate of accumulation, and shorter non-decision time than controls. But patients with progressive supranuclear palsy were strongly biased towards a pro-saccade decision boundary compared to Parkinson’s patients and controls. This indicates a prepotency of responding in combination with a reduction in further accumulation of evidence, which provides a parsimonious explanation for the apparently paradoxical combination of disinhibition and severe akinesia. The combination of the well-tolerated oculomotor paradigm and the sensitivity of the model-based analysis provides a valuable approach for interrogating decision-making processes in neurodegenerative disorders. The mechanistic differences underlying participants’ poor performance were not observable from classical analysis of behavioural data, but were clearly revealed by modelling. These differences provide a rational basis on which to develop and assess new therapeutic strategies for cognition and behaviour in these disorders.

## Teaser

Both progressive supranuclear palsy and Parkinson’s disease cause impulsivity and impair executive function. Using a saccadic Go/No-Go paradigm and hierarchical Bayesian models, Zhang *et al.* show differential decision-making deficits in the two disorders, and that model parameters are better than common behavioural measures for single-patient classification of the diseases.

## Graphical abstract

**Figure:**
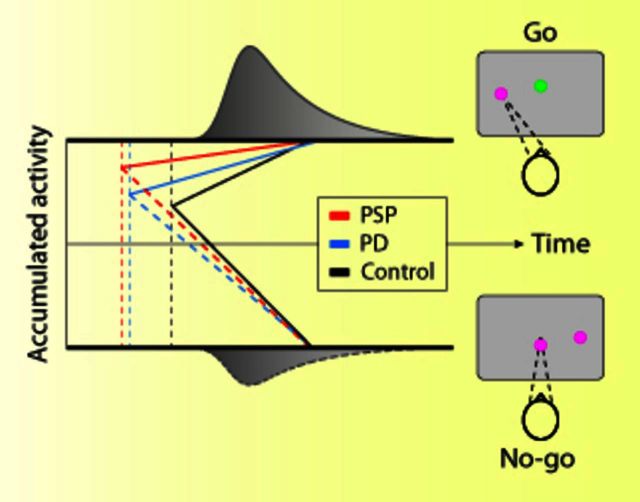
Both progressive supranuclear palsy and Parkinson’s disease cause impulsivity and impair executive function. Using a saccadic Go/No-Go paradigm and hierarchical Bayesian models, Zhang *et al.* show differential decision-making deficits in the two disorders, and that model parameters are better than common behavioural measures for single-patient classification of the diseases.

## Introduction

Parkinson’s disease and progressive supranuclear palsy (PSP) are associated with many non-motor symptoms as well as the motor hallmarks of bradykinesia and rigidity. Despite very different underlying neuropathology ( Hauw *et al.* , 1994 ; Litvan *et al.* , 1996 *b* ; Braak *et al.* , 2003 ), both disorders may lead to executive dysfunction and impulsivity. Impulse control disorders are severe in ∼10% of patients with Parkinson’s disease ( Weintraub *et al.* , 2010 ), but patients are impaired in response inhibition and decision-making even in the absence of impulse control disorders, and also before they are exposed to any dopaminergic therapies ( Obeso *et al.* , 2011 ; Nombela *et al.* , 2014 ; Ye *et al.* , 2014 *a* ). The Richardson’s syndrome or ‘classical’ phenotype of PSP also causes impulsivity despite severe akinesia and apathy ( O’Sullivan *et al.* , 2010 ; Burrell *et al.* , 2014 ). It manifests as delay intolerance, choice impulsivity, and behavioural decisions that increase the risk of falls, and it can exacerbate dysphagia and carer burden ( Wedderburn *et al.* , 2008 ; Burrell *et al.* , 2014 ).

There are several candidate mechanisms by which PSP and Parkinson’s disease may lead to impulsivity and poor response inhibition. For example, loss of subthalamic inhibition would lead to a bias towards action and away from response inhibition, by disinhibition of thalamocortical projections ( Frank *et al.* , 2007 ; Averbeck *et al.* , 2014 ). Cortical neuropathology, especially in prefrontal and premotor circuits ( Braak *et al.* , 2003 ; Brenneis *et al.* , 2004 ; Rae *et al.* , 2012 ) may also impair the appropriate accumulation of evidence for action and inhibition in a given context ( Verbruggen and Logan, 2009 ), and impair the selection and execution of actions ( Zhang *et al.* , 2012 ). Finally, changes in dopaminergic, serotonergic and noradrenergic innervation of the cortex and striatum can influence decision-making and response inhibition ( Ye *et al.* , 2014 *a* , *b* ), while dopaminergic therapies themselves can increase risk-taking behaviours and impulsivity ( Weintraub *et al.* , 2010 ).

The different features of PSP and Parkinson’s disease mean that behavioural deficits in response inhibition may have different origins in these two disorders. In Parkinson’s disease for example, the impulsive choice arising from dopaminergic stimulation is distinct from the impulsive choice arising from therapeutic deep brain stimulation of the subthalamic nucleus ( Frank *et al.* , 2007 ).

**Figure 1:**
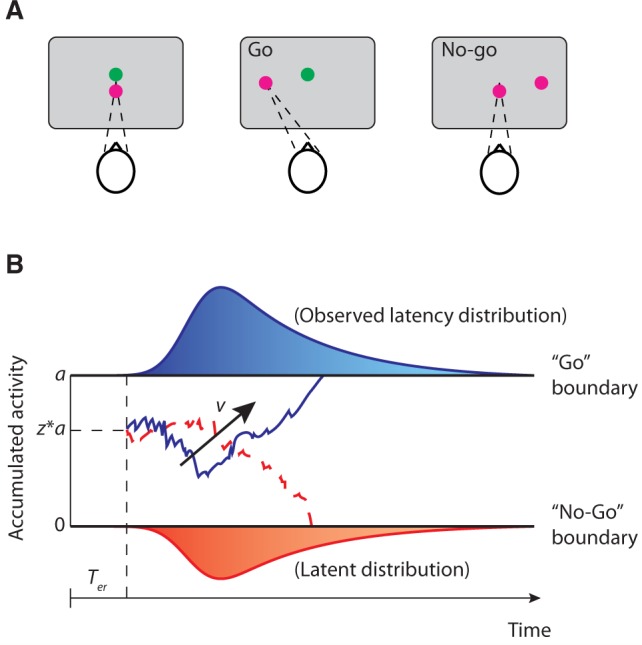
**Saccadic Go/No-Go task and the drift-diffusion model.** ( **A** ) Participants fixated on green and red points overlapping at the centre of the screen. One of the two points disappeared and a saccadic target was presented on the left or right of the screen at an angle of 10° from the fixation point. Participants were instructed to make a saccade to the target if the remaining fixation point was green, or withhold their response if the remaining fixation point was red. ( **B** ) Examples of trajectories of the drift-diffusion model. Two decision boundaries (0 and *a* ) represent the Go and No-Go decisions. The drift rate *v* represents the rate of accumulation. The diffusion process starts at a starting point between the two boundaries ( a*z ) until the accumulated evidence reaches one of the two boundaries. The predicted saccadic latency is the sum of the duration of the diffusion process and the non-decision time *T _er_* .

We tested this hypothesis in two clinically diagnosed patient groups and a healthy control group on a saccadic Go/No-Go task ( Fig. 1 A). The reason for the use of saccadic measures is 2-fold: (i) the precision of the oculomotor system that enables accurate recording and modelling; and (ii) the advantage of measuring the time to initiate movement, not execution time, in patients with bradykinesia.

The underlying cognitive deficits were revealed by formalizing the Go/No-Go task as an accumulation-to-threshold decision process, which can be described by a drift-diffusion model (DDM) ( Gomez *et al.* , 2007 ; Ratcliff and McKoon, 2008 ). This model provides a parsimonious account of complex behavioural phenomena, including response latency distributions ( Ratcliff and Smith, 2004 ), speed accuracy trade-offs ( Zhang and Rowe, 2014 ), and the effects of uncertainty on decision-making ( Mulder *et al.* , 2012 ). The DDM also has direct neurophysiological evidence in support of it, for neurons in the superior colliculus ( Ratcliff *et al.* , 2003 ) and cortex ( Kim and Shadlen, 1999 ; Shadlen and Newsome, 2001 ), while related methods have been used to examine anatomical correlates from functional brain imaging data ( Rowe *et al.* , 2010 ; Zhang *et al.* , 2012 ). This model-based approach has proven valuable and has already revealed the critical role of the subthalamic nuclei for motor inhibition under conditions of ambiguity or risk ( Cavanagh *et al.* , 2011 ) and the effect of subthalamotomy on inhibitory behaviour ( Obeso *et al.* , 2014 ).

Accurate fitting of the model in studies of animals and healthy participants often requires thousands of trials ( Brunton *et al.* , 2013 ). This would not be tolerated by patients with neurodegenerative disorders. We therefore used a Bayesian parameter estimation approach to fit a hierarchical DDM that encompasses patient heterogeneity in terms of random sampling of individuals from a group-wise distribution ( Wiecki *et al.* , 2013 ). By optimizing the model to fit both the distribution of response latencies and the accuracy of responses, the hierarchical DDM is efficient at reproducing behavioural data and the properties of generative decision processes, within a few trials.

The DDM assumes that a single accumulator integrates the momentary evidence over time. This accumulation process terminates when the accumulated evidence reaches an upper or a lower boundary, corresponding to the Go or No-Go decisions ( Fig. 1 B). Impulsive behaviour in the Go/No-Go task can be decomposed into different changes in parameters of the DDM, for example a baseline bias towards ‘Go’ decisions ( Mulder *et al.* , 2012 ), or reduced accumulation rate for ‘No-Go’ decisions ( Obeso *et al.* , 2014 ).

We predicted that both PSP and Parkinson’s disease impair response inhibition but the hierarchical DDM would reveal different causes of these deficits in the two diseases. Knowledge of these mechanisms may not only increase our ability to detect such deficits, but would also enable the development of better mechanistic therapies.

## Materials and methods

### Participants

Sixty-nine participants were recruited. Demographic and clinical features of the participants are summarized in Table 1 . Nineteen patients with PSP were recruited from a regional specialist clinic for PSP and related disorders at the Cambridge University Hospitals NHS Foundation Trust. Consensus clinical diagnostic criteria ( Litvan *et al.* , 1996 *a* ) for probable PSP were used by an experienced neurologist, identifying the Richardson’s syndrome phenotype. To date, 10 of the clinically diagnosed patients have died and been recruited to the Cambridge Brain Bank as part of a separate research programme: all 10 had neuropathological confirmation of the diagnosis.

**Table 1:** Demographics and neuropsychological measures of participants with PSP, Parkinson’s disease and healthy control subjects

	PSP ( *n* = 19)	PD ( *n* = 24)	Control ( *n* = 26)
Age	68.37 ± 6.54	66.17 ± 10.15	66.58 ± 7.10
Gender	8 M / 11 F	13 M / 11 F	10 M / 16 F
MMSE	26.58 ± 3.73	28.29 ± 1.92	29.46 ± 0.95
ACE-R	79.89 ± 12.23	89.63 ± 8.72	94.62 ± 4.05
PSPRS	38.16 ± 16.89	–	–
UPDRS-III	–	33.88 ± 15.89	–

Values are mean ± standard deviation. PD = Parkinson’s disease.

Twenty-four patients with idiopathic Parkinson’s disease (Hoehn and Yahr stage I–III) were recruited through the Cambridge University Parkinson’s Disease Research Clinic. All Parkinson’s patients met the United Kingdom Parkinson’s Disease Society Brain Bank Clinical Diagnostic Criteria ( Gibb and Lees, 1988 ). Patients with Parkinson’s disease did not have pathological confirmation. Additional inclusion criteria were: (i) non-demented at last clinical assessment (Mini-Mental State Examination score, MMSE ≥ 24/30); (ii) no ongoing clinically significant depression (Beck Depression Inventory score ≤ 18; Beck *et al.* , 1988 ).

Twenty-six healthy control participants with no history of significant neurological or psychiatric disorders were recruited from the volunteer panel of the Medical Research Council Cognition and Brain Sciences Unit.

Participants underwent cognitive assessment using the Revised Addenbrooke’s Cognitive Examination (ACE-R) and Mini-Mental State Examination. Disease severity was assessed by the PSP Rating Scale (PSPRS) ( Golbe and Ohman-Strickland, 2007 ) and the Unified Parkinson Disease Rating Scale (UPDRS, part III motor subscale) ( Fahn, 1986 ). All testing was performed with participant’s taking their usual medication. The study was approved by the local research ethics committee. Written informed consent was obtained from all participants.

### Task and procedure

All participants performed a saccadic Go/No-Go task ( Fig. 1 A; Nombela *et al.* , 2014 ). Each participant sat at a distance of 1.5 m from a blank screen wearing a head-mounted saccadometer (Ober consulting) with a binocular infra-red scleral oculometer for measurement of horizontal movements. The infrared reflectance signals were recorded at 1 kHz and low pass filtered at 250 Hz, with 12 bit resolution. Three low-power lasers were mounted on the forehead plate and angled at −10°, 0° and +10° azimuth for stimulus presentation. Because the device and target display moves with the head, a head restraint or a bite bar is not required.

Each experiment session consisted of 300 trials. At the beginning of each trial, the participants fixated on two central spots at 0° (one green, one red). After a random period between 1500 ms and 2500 ms, one of the central spots was extinguished and simultaneously a red target spot was presented with a −10° or +10° horizontal displacement. In 50% of the trials, the green central spot remained and the participants were required to make a saccade to the lateral target (Go trials). In the other 50% of trials, the red central spot remained and the participants were required to hold their saccade and maintain fixation (No-Go trials). The lateral target disappeared 250 ms after a saccade was made, or after a maximum duration of 1500 ms was reached. The order of the target locations (leftwards and rightwards) and the trial type (Go and No-Go) were randomized within and across participants. A short series of 40 presentations of the targets were used at the beginning of the session for calibration.

### Data preprocessing

Eye movement data were downloaded from the saccadometer to a laptop and preprocessed using an automated validation program in Latency Meter 2.3 (Ober consulting). The validation program removed erroneous trials due to blinks, as well as grossly abnormal profiles as determined by the instantaneous velocity, acceleration, and position of eye movement traces, and rejection criteria for either the peak velocity or saccadic duration ( Ober *et al.* , 2003 ).

For trials with valid saccades, the saccadic latency was defined as the time interval between the target onset and the onset of the saccade. The saccadic latency data were pooled for leftward and rightward targets to increase statistical power, because the saccadic latencies did not significantly differ between the two target locations [ *t* (68) = 0.63, *P* = 0.53, paired *t* -test]. Four behavioural measures were obtained for each participant: (i) the rate of omission errors in Go trials; (ii) the rate of commission errors in No-Go trials; (iii) mean saccadic latency in successful Go trials; and (iv) mean saccadic latency in No-Go trials with commission errors.

### Hierarchical drift-diffusion model for the Go/No-Go task

The saccadic Go/No-Go task can be conceptualized as a rapid two-alternative forced choice between a Go decision and a No-Go decision ( Gomez *et al.* , 2007 ). The decision process has been described by a widely accepted DDM ( Ratcliff and McKoon, 2008 ). The model can be described by four parameters ( Fig. 1 B): boundary separation *a* indicating the distance between the two decision boundaries, drift rate *v* indicating the rate of evidence accumulation, *a priori* decision bias *z* indicating the starting point of the accumulator at stimulus onset, and non-decision time *T _er_* indicating the time used for non-decision processes (e.g. stimulus encoding or response execution latencies).

Most decision-making tasks require selecting between two overt responses. For example, in a lexical decision task, participants are instructed to make ‘word’ or ‘non-word’ decisions by pressing one of two response buttons ( Ratcliff *et al.* , 2004 ). In this case, the two decision boundaries in the DDM correspond to the two choice alternatives, and the model predicts the decision time for each choice as the latency of accumulator activity reaching the corresponding boundary. However, the Go/No-Go task differs from the classical binary decision tasks in that response time for a No-Go decision cannot be explicitly measured. In line with previous studies ( Gomez *et al.* , 2007 ), we assumed an implicit lower decision boundary for No-Go decisions and an upper boundary for Go decisions ( Fig. 1 B), and fitted the DDM to individual participant’s responses (i.e. the proportion of Go and No-Go choices) as well as the distributions of saccadic latencies (i.e. in Go trials with successful responses or in No-Go trials with commission errors).

The hierarchical DDM toolbox was used to fit the data ( Wiecki *et al.* , 2013 ). The hierarchical model assumes that participants are random samples drawn from group-level distributions, and uses Bayesian statistical methods to simultaneously estimate parameter distributions at both the group level and the individual-participant level ( Vandekerckhove *et al.* , 2011 ). The Bayesian approach has been shown to be more robust in recording model parameters than other methods such as maximum likelihood estimation when limited data are available ( Jahfari *et al.* , 2013 ). This important feature greatly benefits the current study, because of substantial constraints on the duration of the task for patients.

We examined four variants of the DDM with different parameter constraints. The first model assumed an unbiased starting point ( *z* = 0.5) and the same absolute value for the drift rate in the Go and No-Go conditions (i.e. if the drift rate in the Go condition is *v* , then the drift rate in the No-Go condition is − *v* ). The second model assumed an unbiased starting point but allowed the drift rate to vary between the two conditions. The third model assumed variable starting points across participants and the same absolute value for the drift rate in the two conditions. The fourth model assumed variable starting points and different drift rates in the two conditions. Each model parameter had three group-level distributions corresponding to the three participant groups (PSP, Parkinson’s disease and controls) and individual-level distributions for each participant.

For each model, we generated 15 000 samples from the joint posterior distribution of all model parameters by using Markov chain Monte Carlo methods ( Gamerman and Lopes, 2006 ). The initial 5000 samples were discarded as burn-in to minimize the effect of initial values on the posterior inference (see Wiecki *et al.* , 2013 for more details of the procedure). Geweke statistic was used to assess the convergence of the Markov chains ( Gelman *et al.* , 2004 ). Parameter estimates in all models were converged after 15 000 samples.

### Statistical analysis

ANOVAs and *post hoc t* -tests were used for statistical analysis of the behavioural measures between groups. Statistical inference on model parameters was made by two complementary approaches. First, for each parameter at the individual-participant level, the mean of its posterior distribution was used as a point estimate for comparing between groups. Second, for each parameter at the group level, Bayesian inference was used to directly compare its posterior distribution between groups ( Lindley, 1965 ; Berger and Bayarri, 2004 ; Gelman *et al.* , 2004 ; Kruschke, 2010 ). We use *P* to refer to classical frequentist *P* -values, and *P _p_*_|D_ to refer to the proportion of posteriors supporting the testing hypothesis at the group level from Bayesian inference.

### Model-based classification of individual patients

To investigate how well the model parameters can distinguish between the three participant groups, we used a three-way linear logistic regression classifier implemented in Weka ( http://www.cs.waikato.ac.nz/ml/weka ). For each participant, the feature space for classification included mean estimates of the five model parameters from the best fitted model ( *a* , *z* , *T _er_* , *v _go_* , and *v _no-go_* ). A leave-one-out cross-validation procedure was performed to optimize the use of this limited dataset. In each cross-validation fold, one participant was first removed and the remaining participants’ data were used as a training set to build the classifier. The participant left out was then classified into one of the three groups (PSP, Parkinson’s disease and controls), independently from the training set. Classification performance was evaluated by the hit rate, precision, and the area under the receiver operating characteristic (ROC) curve (AUC) of each class, averaged across all cross-validations.

To assess whether the model parameters provide better discrimination than simple behavioural measures, we performed the same classification procedure with a second feature space, which contained four raw behavioural measures (omission rate, commission rate, mean saccadic latencies in the Go and No-Go conditions).

We used permutation tests to evaluate whether DDM parameters are better than raw behavioural measures in the classification of a participant’s group. Three evaluation criteria were used: weighted average of hit rate across the three classes, weighted average of precision, and weighted average of AUC. The significance of each criterion was determined by comparing the observed evaluation criterion with its distribution under the null hypothesis, which was generated by 100 000 random permutations of leave-one-out classification results between the two feature sets. The permutation *P* -value was then obtained by calculating the probability of the permuted samples exceeding the observed value in the data.

## Results

### Behavioural results

Details of participant demographics, disease severity and neuropsychological scores are given in Table 1 . The three groups were well matched for age [ *F* (2,68) = 0.42, *P =* 0.66] and gender ( *P* = 0.51, chi-square test). As expected, cognitive performance differed significantly between groups [MMSE: *F* (2,68) = 8.41, *P* < 0.001; ACE-R: *F* (2,68) =16.34, *P* < 0.00001]. Both patient groups had lower MMSE scores than controls [PSP: *t* (43) = −3.79, *P* < 0.001; Parkinson’s disease: *t* (48) = −2.76, *P* < 0.01] and total ACE-R scores [PSP: *t* (43) = −5.74, *P* < 0.00001; Parkinson’s disease: *t* (48) = −2.63, *P* < 0.05]. Patients with PSP also had lower ACE-R [ *t* (41) = −3.04, *P* < 0.01] and marginally lower MMSE [ *t* (41) = −1.95, *P =* 0.06] than patients with Parkinson’s disease.

**Figure 2:**
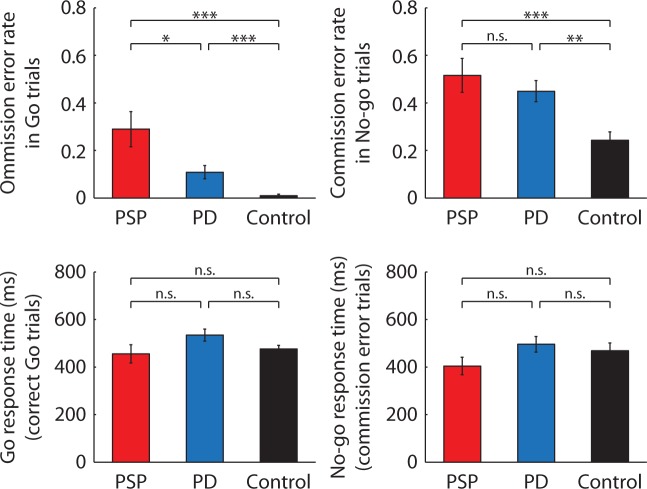
**Behavioural results.** The mean proportion of errors ( *top* ) and saccadic latencies ( *bottom* ) in the Go/No-Go task. The error bars represent the standard errors across participants in each group. In all panels, asterisks denote statistical significance at * *P* < 0.05, ** *P* < 0.01, or *** *P* < 0.001 from independent sample *t* -tests and n.s. denotes non-significant difference. PD = Parkinson’s disease.

Behavioural results are shown in Fig. 2 . There were significant group differences in the omission error in the Go condition [ *F* (2,68) = 12.37, *P* < 0.0001; partial *η*^2^ = 0.27], and in the commission error in the No-Go condition [ *F* (2,68) = 7.20, *P* < 0.001; partial *η*^2^ = 0.18]. Compared with controls, both patient groups had higher omission errors [PSP: *t* (43) = 4.45, *P* < 0.0001; Parkinson’s disease: *t* (48) = 3.58, *P* < 0.001] and higher commission errors [PSP: *t* (43) = 3.42, *P* < 0.001; Parkinson’s disease: *t* (48) = 3.27, *P* < 0.01]. Patients with PSP had higher omission errors than patients with Parkinson’s disease [ *t* (41) = 2.49, *P* < 0.05], and the two patient groups had similar commission errors [ *t* (41) = 0.83, *P* = 0.41]. There was no significant group difference in the saccadic latency in successful Go trials [ *F* (2,68) = 2.34, *P* = 0.10; partial *η*^2^ = 0.07] or No-Go trials with commission errors [ *F* (2,68) = 1.65, *P* = 0.20; partial *η*^2^ = 0.05].

### Hierarchical drift-diffusion model fit to saccadic Go/No-Go data

We compared four variants of the hierarchical DDM for the saccadic Go/No-Go task, varying systematically in constraints on whether the starting point was biased towards one of the two decision boundaries, and whether the drift rate varied between Go and No-Go conditions, because the drift rate is often assumed to change between stimulus conditions ( Gomez *et al.* , 2007 ). For each model, a Bayesian parameter estimation procedure was used to estimate the joint posterior distributions of all the model parameters, given the observed behavioural data. To identify the model with the best fit, we estimated the deviance information criterion (DIC) value of each model, a goodness-of-fit measure for Bayesian models with a penalty for additional free model parameters ( Spiegelhalter *et al.* , 2002 ).

**Figure 3:**
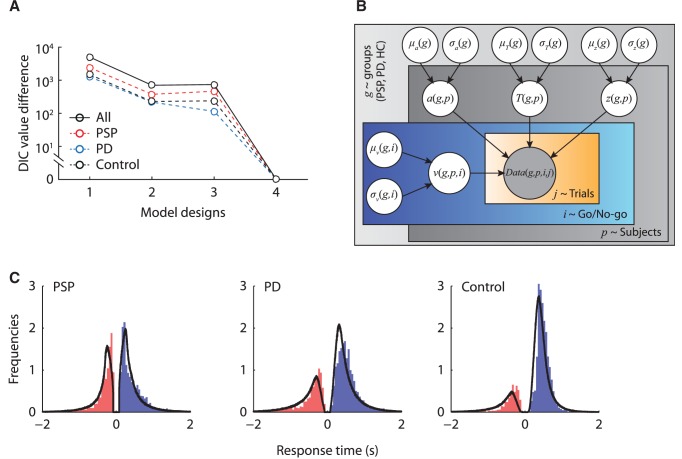
**Model comparison and model fits.** ( **A** ) The deviance information criterion (DIC) value differences between the best fit model (Model 4) and the other three model variants, for each group separately (dash lines) and all participants combined (solid lines). ( **B** ) The graphical representation of the best fit model. The shaded node Data( *g,p,i,j* ) indicates the observed data of each group ( *g* ), participant ( *p* ), condition ( *i* ) and trial ( *j* ). Nodes *a* , *T _er_ , z, and v* are parameters of the drift-diffusion model, each with a group distribution for each patient group with mean *µ* and standard deviation *σ* . ( **C** ) Posterior predictive data distributions from the best fit model. The distribution along the positive *x* -axis shows the latency distribution in the Go condition (correct Go trials), and the distribution along the negative *x* -axis shows the latency distribution in the No-Go condition (commission error trials). Each panel shows the normalized histograms of the observed data and the model prediction (black lines). The area under the curve on the positive *x* -axis corresponds to the observed and predicted accuracy in the Go condition. The area under the curve along the negative *x* -axis corresponds to the commission error in the No-Go condition. PD = Parkinson’s disease.

The best model (with the lowest DIC value) to describe the data across Go/No-Go conditions and participants had a variable starting point between participants and variable drift rates between Go and No-Go conditions (Model 4 in Fig. 3 A, see also Fig. 3 B). To evaluate the model fit, we compared posterior model predictions with the observed data. The posterior predictions of the best model were generated by averaging 500 simulations of the same amount of model predicted data as observed in the experiment using posterior parameter estimates. There was a good agreement between the observed data and the model predictions across conditions in all three participant groups ( Fig. 3 C).

### Inferences from model parameters

**Figure 4:**
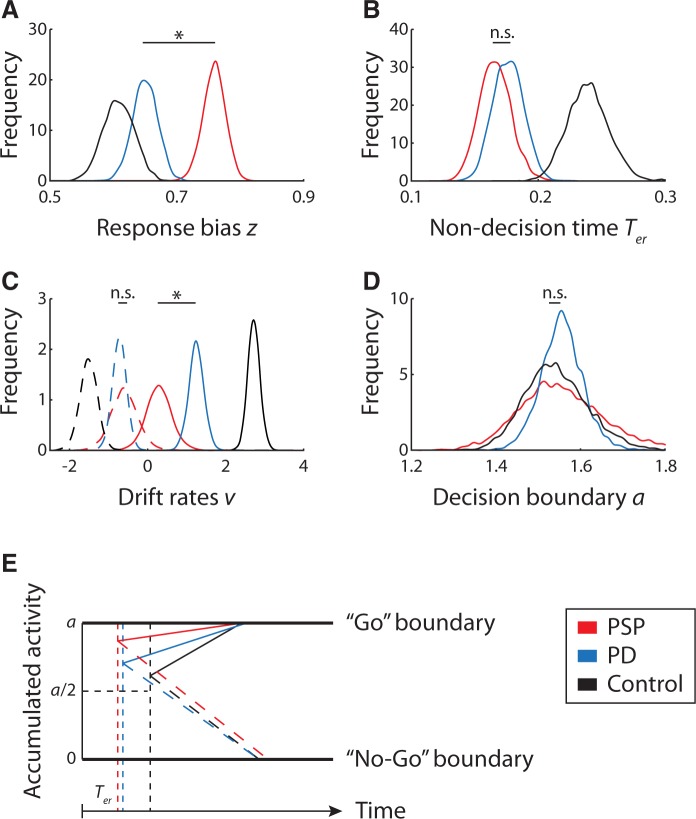
**Posterior estimates of the hierarchical drift-diffusion model parameters for each group.** ( **A** ) Response bias *z* . ( **B** ) Non-decision time *T _er_* . ( **C** ) Draft rates *v* for Go (solid lines) and No-Go (dashed lines) conditions. ( **D** ) Boundary separation *a* . In all panels, the asterisks denote significant difference between PSP and patients Parkinson’s disease from frequentist and Bayesian statistics, and n.s. denotes non-significant difference. ( **E** ) The schematic diagram of the altered Go/No-go decision processes in patients. PSP leads to an exaggerated response bias towards Go decisions (i.e. the upper boundary), and a reduced non-decision time, but that the further accumulation of evidence towards a response is accumulated very slowly, predisposing patients to inhibition errors but without prolonged latencies of actual responses. In contrast, Parkinson’s disease leads to a shorter non-decision time but normal initial response bias and a mild reduction in the rate of accumulation of evidence. PD = Parkinson’s disease.

Figure 4 shows the posterior parameter estimates for the three participant groups. We used both frequentist and Bayesian statistics to examine group differences in model parameters.

#### Response bias

The starting point was significantly larger than 0.5 in all three groups [PSP: *t* (18) = 13.10, *P* < 1 × 10 ^−9^ , *P*_P|D_ ≈ 1; Parkinson’s disease: *t* (23) = 7.85, *P* < 1 × 10 ^−7^ , *P*_P|D_ ≈ 1; control: *t* (25) = 9.52, *P* < 1 × 10 ^−9^ , *P*_P|D_ ≈ 1], indicating that there is a prepotent bias towards the Go response (i.e. the upper decision boundary). However, the absolute magnitude was small in healthy controls and we observed a significant difference in the response bias between groups [ *F* (2,68) = 18.16, *P* < 0.000001, partial *η*^2^ = 0.36]. No significant difference was observed between patients with Parkinson’s disease and controls [ *t* (48) = 1.28, *P* = 0.21, *P*_P|D_ = 0.86]. The PSP group had a significantly larger bias towards the Go response than the Parkinson’s group [ *t* (41) = 4.20, *P* < 0.001, *P*_P|D_ ≈ 1] and controls [ *t* (43) = 6.14, *P* < 0.000001, *P*_P|D_ ≈ 1], indicating that despite their difficulty in moving because of akinetic rigidity, patients with PSP are actually close to the movement threshold.

#### Accumulation of evidence for the response

There was a significant group difference in the drift rate in both Go and No-Go conditions [Go: *F* (2,68) = 36.68, *P* < 1 × 10 ^−10^ , partial *η*^2^ = 0.53; No-Go: *F* (2,68) = 4.38, *P* < 0.05, partial *η*^2^ = 0.12]. The drift rate in the Go condition was lower in the PSP group than that in the Parkinson’s disease group [ *t* (41) = 2.96, *P* < 0.01, *P*_P|D_ = 0.99] and controls [ *t* (43) = 6.54, *P* < 1 × 10 ^−7,^*P*_P|D_ ≈ 1], whereas the patients with Parkinson’s disease also had a lower Go drift rate than the controls [ *t* (48) = −6.54, *P* < 1 × 10 ^−7^ , *P*_P|D_ ≈ 1]. For the No-Go condition, both patient groups had lower drift rate than controls [PSP: *t* (43) = −2.33, *P* < 0.05, *P*_P|D_ = 0.98; Parkinson’s disease: *t* (48) = −3.02, *P* < 0.01, *P*_P|D_ = 0.99], but no significant difference was observed between the two patient groups [ *t* (41) = 0.25, *P =* 0.80, *P*_P|D_ = 0.61].

#### Non-decision time and boundary separation

The three participant groups significantly differed in their non-decision time [ *F* (2,68) = 15.63, *P* < 0.00001, partial *η*^2^ = 0.32]. Both patient groups had a shorter non-decision time than controls [PSP: *t* (43) = −4.54, *P* < 0.0001, *P*_P|D_ ≈ 1; Parkinson’s disease: *t* (48) = −4.30, *P* < 0.0001, *P*_P|D_ ≈ 1], and the non-decision time was similar between the two patient groups [ *t* (41) = 0.89, *P* = 0.38, *P*_P|D_ = 0.74]. The three participant groups had similar boundary separation between thresholds for Go and No-Go decisions [ *F* (2,68) = 0.14, *P* = 0.87, partial *η*^2^ = 0.004].

### Model-based classification

**Figure 5:**
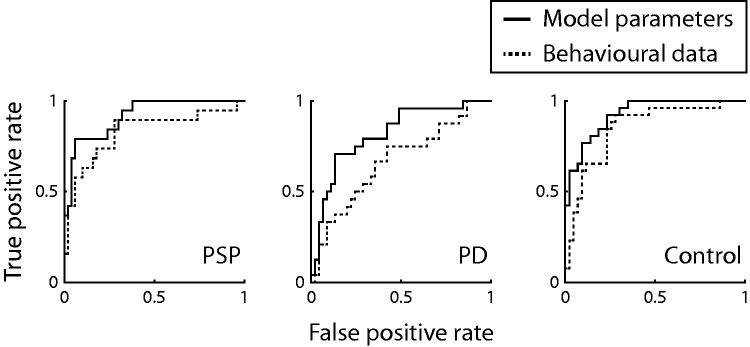
**Receiver operating characteristic curves of each class in the leave-one-out three-way classification based on the model parameters (solid lines) and raw behavioural measures (dash lines).** PD = Parkinson’s disease.

We used a leave-one-out cross validation procedure in a three-way classification of participant groups (PSP, Parkinson’s disease, and controls). First we used the four raw behavioural measures (commission error, omission error, saccadic latency distributions in the Go and No-Go conditions) as the feature space. As expected from the descriptive statistics and group contrasts, there was only a modest ability to classify participants, such that 50–60% of patients were correctly classified ( Table 2 ). The associated ROC curves illustrate the limited performance of behavioural measures, and in particular, show limited discrimination of the two patient groups ( Fig. 5 ).

**Table 2:** Leave-one-out cross-validation results from three-way linear logistic regression classifiers

Feature sets	True positive rate				Precision				AUC of ROC curves			
	PSP group	PD group	Control group	Weighted average	PSP group	PD group	Control group	Weighted average	PSP group	PD group	Control group	Weighted average
DDM parameters	0.73	0.67	0.85	0.75	0.82	0.73	0.73	0.76	0.92	0.81	0.93	0.89
Behavioural measures	0.63	0.50	0.77	0.64	0.75	0.55	0.65	0.64	0.82	0.66	0.85	0.78
Permutation *P* -values for DDM superiority				*P* < 0.05				*P* = 0.07				*P* < 0.0001

Permutation tests were used to compare the classification results based on DDM parameters and raw behavioural measures. For classification based on DDM parameters, three PSP patients were misclassified as controls and two as Parkinson’s disease (PD) patients; while five patients with Parkinson’s disease were misclassified as controls and three as patients with PSP. For classification based on behavioural measures, two patients with PSP were misclassified as controls and five as patients with Parkinson’s disease; whereas nine patients with Parkinson’s disease were misclassified as controls and three as patients with PSP.

We then used the model parameters as a second feature space for classification using the same cross-validation procedure. The model-based approach showed superior sensitivity and precision in classification across the three groups (>75%) ( Table 2 ). The accompanying ROC curves illustrate the significant enhanced ability of model parameters to differentiate participants ( Fig. 5 ). The significant improvement in classification using model parameters over raw behavioural data was confirmed with permutation tests.

## Discussion

We confirmed the impairment of response inhibition in patients with PSP and Parkinson’s disease, but identified significant differences in these disorders on the decision processes that lead to disinhibition. Both patient groups made more commission errors despite akinesia ( O’Sullivan *et al.* , 2010 ; Nombela *et al.* , 2014 ). However, the striking result in PSP was that patients were strongly biased towards making a response and yet were severely impaired at accumulating the necessary evidence to commit to that response ( Fig. 4 E). This combination provides a parsimonious explanation for the apparently paradoxical combination of impulsivity and akinesia seen in this disease.

The study highlights the benefits of formal computational modelling of behaviour for a better understanding of disease mechanisms. Bayesian parameter estimation of the drift-diffusion model provided a highly efficient and robust measure of an individual’s performance, with many fewer data needed in comparison with other approaches in preclinical and normative studies ( Vandekerckhove *et al.* , 2011 ; Wiecki *et al.* , 2013 ; Zhang and Rowe, 2014 ). This approach enables one to infer disease-specific changes at the group level.

The model decomposed behavioural data into five parameters associated with Go/No-Go decisions: response bias, drift rates in Go and No-Go conditions, boundary separation and non-decision time. These model parameters also improved the discrimination of patients and controls over the common behavioural measures (errors and reaction times), with higher precision and superior signal detection in classification (as receiver operating characteristics). Simple behavioural measures, like the mean latency of response, have been examined before in both diseases, but with mixed conclusions. In patients with Parkinson’s disease, pro-saccades may have longer latency to controls ( Michell *et al.* , 2006 ) or normal ( Chan *et al.* , 2005 ; van Koningsbruggen *et al.* , 2009), and reflective saccades may be faster ( Briand *et al.* , 2001 ). Horizontal saccadic latency in PSP has been reported to be either slower than controls ( Linder *et al.* , 2012 ; Ghosh *et al.* , 2013 ) or normal, at least at the group level ( Pierrot-Deseilligny *et al.* , 1989 ; Vidailhet *et al.* , 1994 ). By using the full distribution of response latencies and accuracy, the model-based approach instead provides clear evidence of abnormality in both PSP and Parkinson’s disease. This result implies that data analysis methods for disease monitoring or drug response monitoring need to be more sophisticated than basic behavioural measures. The effect of a candidate drug on behaviour may be missed if crude metrics like reaction time alone are used. Our modelling approach has greater potential to support clinical trials.

The model-based analysis revealed that behavioural impairments of response inhibition in PSP and Parkinson’s disease arise for different reasons. This implies that effective treatment strategies for one disease may not work for the other. However, in both patient groups and controls, a response bias towards Go decisions was consistently observed. This could be explained by the reactive nature of Go saccades and an underlying bias in response neuron populations. For example, neurons in the intermediate layer of the superior colliculus have stronger sustained activities in Go trials than No-Go trials ( Paré and Wurtz, 2001 ), which might enhance descending supranuclear control for saccades.

Patients with PSP demonstrated more severe response bias than patients with Parkinson’s disease and control subjects, which could be explained by several pathophysiological mechanisms. Abnormal ocular fixation, such as square wave jerks, is more apparent in PSP ( Garbutt *et al.* , 2004 ; Otero-Millan *et al.* , 2011 ) than in Parkinson’s disease ( Rascol *et al.* , 1991 ). This has been attributed to midbrain atrophy in PSP ( Kato *et al.* , 2003 ). The disease interrupts inputs to omnipause neurons (e.g. from rostral superior colliculus, see Everling *et al.* , 1998 ), which in turn changes the reciprocal discharge patterns of omnipause neurons and burst neurons in the pontine reticular formation. At the behavioural level, the affected brainstem circuitry would be prone to initiate saccades, leading to a strong bias towards Go responses in the Go/No-Go task.

It is also worth considering the contribution of cortical pathology. ^18^ F-fluorodeoxyglucose PET imaging has identified decreased metabolic activity in the medial prefrontal cortex, anterior cingulate, and ventrolateral prefrontal cortex in PSP ( Eckert *et al.* , 2008 ). These regions are part of the cortical network essential for executive control ( Ridderinkhof *et al.* , 2004 ) and response inhibition of both eye and hand movements ( Leung and Cai, 2007 ). Therefore severe response bias in PSP may also result from cortical degeneration, which imbalances the binary decision between Go and No-Go choices ( Mulder *et al.* , 2012 ).

Patients’ with PSP and Parkinson’s disease had slower drift rates than controls in both Go and No-Go conditions. This result suggests the effects of the diseases on the accumulation of decision signal: prolonging the latency to reach a decision boundary and thereby increasing response errors. Previous work on Parkinson’s disease is consistent with this account. Briand *et al.* (1999) showed longer saccadic latency in patients with Parkinson’s disease in an anti-saccade task ( Antoniades *et al.* , 2013 ). Similar results were reported in studies using pro-saccade paradigms ( Amador *et al.* , 2006 ; Michell *et al.* , 2006 ) and manual reaction time tasks ( Gauntlett-Gilbert and Brown, 1998 ). Using voxel-based morphometry, Perneczky *et al.* (2011) identified that the longer and more variable saccadic latency in patients with Parkinson’s disease was associated with lower grey matter volume of the frontal eye field and lateral prefrontal cortex. The frontal-subcortical pathway plays a central role for the generation of saccades with precise timing ( Robinson and Fuchs, 2001 ), and the accurate saccade control in response to different task demands via the prefrontotectal tract ( Robinson and Fuchs, 2001 ). In Parkinson’s disease, cortical and subcortical atrophy disrupts this saccadic decision network ( Tinaz *et al.* , 2011 ; Rae *et al.* , 2012 ), which may give rise to the lowered drift rates observed in the current study. Similarly, prolonged pro-saccade latencies have also been reported in PSP ( Ghosh *et al.* , 2010 ). We speculate that this is also caused by atrophy in saccadic control regions ( Ghosh *et al.* , 2012 ), which is yet to be confirmed in imaging studies that correlate with saccade latency.

An intriguing finding is that patients with PSP and those with Parkinson’s disease had shorter non-decision time than controls. The non-decision time reflects the latency of early sensory encoding external to the oculomotor decision process ( Ratcliff and McKoon, 2008 ; Wagenmakers, 2009 ). Therefore, shorter non-decision time could imply enhanced early sensory processing in patients. This result may at first seem surprising, given motor akinesia and cognitive slowing associated with the diseases. Nevertheless, our result is consistent with EEG evidence. In a visual oddball paradigm, the latency of the early event-related potential N1 in patients with Parkinson’s disease was shorter than that in healthy control subjects ( Wang *et al.* , 2001 ; Li *et al.* , 2003 ), suggesting excessive attention or enhanced sensory processing in patients. This concurs with the hypothesis ( Palop *et al.* , 2006 ) that impaired cognitive function in neurodegenerative disorders can be compensated for by additional processing, such as increased reliance on visual features ( Bloem *et al.* , 2004 ; Helmich *et al.* , 2007 ).

In both PSP and Parkinson’s disease there is abundant evidence for impulsive limb movements and global behaviours ( Litvan *et al.* , 1996 *a* ; Aarsland *et al.* , 2001 ; Robert *et al.* , 2009 ; O’Sullivan *et al.* , 2010 ; Jahanshahi *et al.* , 2014 ; Nombela *et al.* , 2014 ). For example, patients with Parkinson’s disease have higher commission errors than controls in manual Go/No-Go tasks ( Nombela *et al.* , 2014 ), and longer stop-signal reaction time in manual stop-signal tasks ( Gauggel *et al.* , 2004 ; Ye *et al.* , 2014 *a* ). Therefore the failure of response inhibition is not restricted to eye movements as studied here. It is possible that the origin of limb inhibition deficits is different to oculomotor inhibition deficits, but we propose that the two types of decision deficit are homologous. Several lines of research support this hypothesis. For instance, limb kinetics affect saccadic outputs in health ( Snyder *et al.* , 2002 ; van Donkelaar *et al.* , 2004), and both are comparably impaired in Parkinson’s disease ( Gibson *et al.* , 1987 ). Onset latencies for eye and hand movements are correlated in many tasks ( Lunenburger *et al.* , 2000 ; Sailer *et al.* , 2000 ; Gribble *et al.* , 2002 ; Snyder *et al.* , 2002 ), including in stop-signal tasks ( Boucher *et al.* , 2007 ). Furthermore, the same accumulator model, assuming competitions between a Go process and a Stop process during response inhibition, provides a good fit for data from both saccadic and manual stop-signal tasks ( De Jong *et al.* , 1990 ; Hanes *et al.* , 1998 ; Gopal and Murthy, 2015 ). Therefore, although the inhibited movements of saccades and manual movements are not controlled by an identical anatomical pathway, different inhibitory systems may share the same computational principals: disruption to this process therefore gives rise to similar impulsivity across response modalities in diseases. This account is consistent with the findings that deep brain stimulation in Parkinson’s disease influences inhibitory controls over saccadic as well as manual responses (van den Wildenberg *et al.* , 2006; Yugeta *et al.* , 2010 ; Swann *et al.* , 2011 ; Jahanshahi, 2013 ).

There are several limitations to this study. First, the severe response bias towards Go decisions indicates that PSP is associated with impulsivity in saccadic inhibition. However, impulsivity is a multi-modal construct ( Dalley *et al.* , 2011 ) and our study alone does not show whether performance impairments in behavioural paradigms such as the Go/No-Go task are associated with different domains of cognition and impulsivity. Saccadic latencies target impaired decision processes in the cortical and subcortical supranuclear network and cognitive precursors to oculomotor inhibition. This is not necessarily a sensitive measure of a broad range of other higher-order cognitive deficits in Parkinson’s disease and PSP ( Burrell *et al.* , 2014 ; Yarnall *et al.* , 2014 ). Nevertheless, saccadic control involves widespread cortico-striato-thalamo-cortical circuits that are essential to cognition ( Alexander *et al.* , 1990 ; Hikosaka *et al.* , 2000 ), which makes saccades a valuable tool for understanding cognitive dysfunctions ( Leigh and Kennard, 2004 ). A recent study on the fractionation of impulsivity provides promising results in this regard. Commission errors in the saccadic Go/No-Go task, together with other tasks with demands on conflict resolution, are associated with the self-assessment of impulsive behaviours on the Barrett Impulsivity Scale ( Nombela *et al.* , 2014 ), suggesting the sensitivity and broad relevance of saccadic tasks in relation to clinical features.

Second, we have reported that model parameters were more informative than the commonly derived behavioural measures (mean latencies and errors) in classification. This does not mean that the model parameters are more informative than the raw data, but reflects the fact that the model parameters are sensitive to higher order moments of the reaction time distributions, especially the skew, kurtosis and variance. Addition of these higher order moments might improve accuracy of classification, but would not provide the mechanistic interpretation of the deficits as revealed from the DDM.

Finally, the patients were recruited from a regional clinic and therefore may not fully represent the whole population of patients at different disease stages. We included a modest number of patients in each group, which was sufficient for detecting disease-specific differences. Given the fact that saccadometry is well tolerated in patients, our protocol could be extended to a larger cohort, from which the increased statistical power would allow one to further investigate the association between model parameters and clinical measures. Interestingly, a recent longitudinal study showed that, in PSP, oculomotor function and cognition were affected early in the course of the illness ( Ghosh *et al.* , 2013 ). Therefore, although our approach is potentially useful to explore the effects of treatment or disease on oculomotor and decision-making systems, model-based analysis of longitudinal data would be required to identify appropriate biomarkers for tracking disease progression in individual patients.

In conclusion, impairments of saccadic response inhibition occur in both PSP and Parkinson’s disease. Both diseases impaired information sampling during decision-making, while patients with PSP showed an additional stronger, disease-specific bias towards Go decisions. We further demonstrated that computational modelling is more efficient than raw behavioural measures when used for discriminating between patients. These results have the potential to be exploited in future diagnostic and therapeutic studies for the comprehensive understanding of different disease mechanisms and in the evaluation of disease-modifying treatments.

## Funding

This work was supported by Medical Research Council (MC-A060-5PQ30 and clinical training fellowship G1100464 to T.R.); Wellcome Trust (Senior fellowship to J.B.R. 103838 and joint award with MRC to the BCNI); Beverly Sackler scholarship to T.R.; the NIHR Cambridge Biomedical Research Centre including the Cambridge Brain bank; Parkinson’s UK; and the James F McDonnell Foundation 21st century science initiative on Understanding Human Cognition. A.F. was supported by University of Cambridge and East Anglia Foundation School.

## Abbreviations

DDM: drift-diffusion model
PSP: progressive supranuclear palsy
